# Infectious pathogens and risk of oesophageal, gastric and duodenal cancers and ulcers in China: a case-cohort study

**DOI:** 10.1002/ijc.34814

**Published:** 2023-12-18

**Authors:** Christiana Kartsonaki, Pang Yao, Julia Butt, Rima Jeske, Catherine de Martel, Martyn Plummer, Dianjianyi Sun, Sarah Clark, Robin G. Walters, Yiping Chen, Jun Lv, Canqing Yu, Michael Hill, Richard Peto, Liming Li, Tim Waterboer, Zhengming Chen, Iona Y. Millwood, Ling Yang

**Affiliations:** 1 Clinical Trial Service Unit & Epidemiological Studies Unit (CTSU), Nuffield Department of Population Health, University of Oxford, Oxford, UK; 2 Medical Research Council Population Health Research Unit (MRC PHRU), Nuffield Department of Population Health, University of Oxford, Oxford, UK; 3 Infections and Cancer Epidemiology Division, German Cancer Research Center (DKFZ), Heidelberg, Germany; 4 Early Detection, Prevention and Infections Branch, International Agency for Research on Cancer, Lyon, France; 5 Department of Statistics, University of Warwick, Coventry, UK; 6 Department of Epidemiology & Biostatistics, School of Public Health, Peking University, Xueyuan Road, Haidian District, Beijing 100191, China; 7 Peking University Center for Public Health and Epidemic Preparedness and Response, Beijing 100191, China

## Abstract

Infection by certain pathogens is associated with cancer development. We conducted a case-cohort study of ~2500 incident cases of oesophageal, gastric, and duodenal cancer, and gastric and duodenal ulcer and a randomly selected subcohort of ~2000 individuals within the China Kadoorie Biobank study of >0.5 million adults. We used a bead-based multiplex serology assay to measure antibodies against 19 pathogens (total 43 antigens) in baseline plasma samples. Associations between pathogens and antigen-specific antibodies with risks of site-specific cancers and ulcers were assessed using Cox regression fitted using the Prentice pseudo-partial likelihood.

Seroprevalence varied for different pathogens, from 0.7% for Hepatitis C virus (HCV) to 99.8% for Epstein−Barr virus (EBV) in the subcohort. Compared with participants seronegative for the corresponding pathogen, *H. pylori* seropositivity was associated with a higher risk of non-cardia (adjusted hazard ratio [HR] 2.73 [95% CI 2.09-3.58]) and cardia (1.67 [1.18-2.38]) gastric cancer, and duodenal ulcer (2.71 [1.79-4.08]). HCV was associated with a higher risk of duodenal cancer (6.23 [1.52-25.62] and Hepatitis B virus was associated with higher risk of duodenal ulcer (1.46 [1.04-2.05]). There were some associations of antibodies again some herpesviruses and human papillomaviruses with risks of gastrointestinal cancers and ulcers but these should be interpreted with caution.

This first study of multiple pathogens with risk of gastrointestinal cancers and ulcers demonstrated that several pathogens are associated with risks of gastrointestinal cancers and ulcers. This will inform future investigations into the role of infection in the aetiology of these diseases.

## Introduction

Chronic infections with certain viruses and bacteria play an important role in the aetiology of cancer, causing about 2.2 million new cancer cases in 2018, with low- and middle-income countries such as China particularly affected.^1–4^ *Helicobacter pylori* (*H. pylori*), Human Papillomaviruses (HPV), Hepatitis B and C viruses (HBV, HCV), and Epstein−Barr virus (EBV) are the most important known cancer-causing pathogens, accounting for substantial proportions of gastric, cervical, and liver cancer, and lymphoma worldwide, respectively.^4^ *H. pylori* infection is a well-established cause of peptic ulcer and gastric cancer,^5^ responsible for >300,000 cases of gastric cancer in China in 2018.^6^ There is also epidemiological evidence that HBV and EBV are associated with gastric cancer.^7,8^

However, there are not many prospective studies on the associations of various infectious pathogens with risks of certain types of gastrointestinal cancers, or ulcers, especially in China where there are high incidence rates of infection-related cancers.^4^ Although the association of *H. pylori* infection with gastric cancer has been well-studied,^5,6,9^ previous studies have not investigated comprehensively the relationships between other pathogens and cancers at different sites along the gastrointestinal tract, as well as ulcers. Moreover, some studies had a retrospective design which may affect seropositivity to certain pathogens.

The aim of this study was to assess the associations between seropositivity for 19 infectious pathogens, measured using a multiplex serology assay, with risks of incident oesophageal, gastric, and duodenal cancers and ulcers in a case-subcohort study within a large cohort of Chinese adults.

## Methods

### Study population and data collection

The China Kadoorie Biobank (CKB) is a prospective cohort study of 512,715 Chinese adults.^10^ 210,205 men and 302,510 women aged 30–79 years were recruited into the study from 10 areas (5 urban and 5 rural) in China during 2004–2008. International, national, and regional ethical approvals were obtained, and all participants provided written informed consent.

At local study assessment clinics, participants completed an interviewer-administered laptop-based questionnaire on sociodemographic characteristics, smoking, alcohol consumption, diet, tea drinking, physical activity, personal and family medical history, and current medication. A range of physical measurements, (e.g. anthropometry, blood pressure and lung function) were recorded by trained technicians, using calibrated instruments with standard protocols. A 10-ml non-fasting (with the time since the participant last ate recorded) blood sample was collected from participants into an EDTA vacutainer (BD Hemogard™, USA).

### Mortality and morbidity follow-up

The vital status of each participant was determined periodically through the Disease Surveillance Points (DSP) system of China CDC,^11^ supplemented by regular checks against local records. In addition, information about occurrence of major diseases and any episodes of hospitalisation was collected through linkage, via each participant’s unique national identification number, with disease registries and national health insurance claims databases. All events were coded using International Classification of Diseases 10th Revision (ICD-10) by trained staff who were blinded to baseline information.

By January 1^st^, 2017, 44,037 (8.6%) participants had died, 4,781 (0.9%) were lost to follow-up and 27,903 (5.4%) had developed cancer, including 2,507 (0.5%) oesophageal cancer (ICD-10 C15), 3,464 (0.7%) gastric cancer (C16, among which 535 were cardia gastric cardia cancer [C16.0]), and 107 (0.02%) duodenal cancer (C17.0) cases; 2,911 (0.6%) participants had developed gastric ulcers (K25) and 1,154 (0.2%) had duodenal ulcers (K26). Systematic cancer validation and adjudication is ongoing, with retrieval of original medical records from hospitals for any reported cancer cases to confirm the cancer diagnosis along with collection of detailed clinical diagnosis information e.g. cancer sub-site, histopathological subtype, stage and grade. Among the ~19000 cancer cases that have been validated in CKB, the overall accuracy of gastric cancer diagnosis was 92% (85% were of adenocarcinoma subtype) and 96% for oesophageal cancer diagnosis (88.5% were squamous cell carcinoma).

### Case-cohort study

A case-cohort study design was used for the present study (eFigure 1). Among all gastric cancer cases recorded at least 2 years after the start of follow-up and up to January 1^st^, 2017 with an available plasma sample and no history of cancer, we selected all 437 recorded cardia gastric cancer (CGC) cases and randomly selected 500 non-cardia gastric cancer (NCGC) cases from 762 validation-confirmed cases. We included all 27 individuals with a validated diagnosis of oesophageal adenocarcinoma (EAC), 500 other oesophageal cancer cases, and all 70 reported duodenal cancer cases. 300 gastric ulcer and 200 duodenal ulcer cases were selected at random from 1900 gastric ulcer and 790 duodenal ulcer reported cases, respectively, that occurred after the amended baseline and did not have any cancer diagnosis in the two years after ulcer diagnosis or any time before ulcer diagnosis. A subcohort of 2000 participants was sampled using simple random sampling from the ‘modified baseline’ cohort (surviving individuals with no history of cancer 2 years after entering the study who had an available plasma sample and had genotyping data available as part of a random sample of the cohort which had been selected for genotyping).

### Multiplex assay

Stored baseline plasma samples of 3950 participants were assayed with a custom-designed multiplex serology assay using a Luminex bead-based method, as described previously.^12,13^ This semi-quantitative assay measures the median fluorescence intensity (MFI), which corresponds to the levels of antibodies against an antigen of interest in a plasma sample.

We measured antibody levels against 43 antigens from 19 pathogens (eTable 1), including herpesviruses (herpes simplex viruses 1 [HSV-1] and 2 [HSV-2], varicella zoster virus [VZV], EBV, cytomegalovirus [CMV], human herpesviruses 6 [HHV-6] and 7 [HHV-7]), HBV and HCV, human papillomaviruses 16 (HPV-16) and 18 (HPV-18), human polyomaviruses (BK, JC, MCV), human immunodeficiency virus (HIV), human T lymphotropic virus type 1 (HTLV-1), *Chlamydia trachomatis* (*C. trachomatis*), *Toxoplasma gondii* (*T. gondii*) and *H. pylori*.^13^ These 19 pathogens were either established or potential risk factors for cancer, or cardiovascular or neurodegenerative diseases, or are of novel scientific interest. Antigen-specific cut-offs to define seropositivity were used as described previously,^14^ and were quality assured by a visual inflection point method.^15^ The definitions for seropositivity for each pathogen are shown in eTable 1.

### Statistical analysis

Of the 3950 participants assayed, after exclusions for assay or sample issues (n=5), 3945 individuals (1964 cases and 1986 subcohort members) remained in the main analyses. Histograms were plotted to visually inspect the distributions of antibody levels to each antigen. We calculated Kendall’s correlations between MFI levels of antibodies and Pearson’s correlations between the seropositivity of pathogens.

The associations between seropositivity for each pathogen and each antigen with risk of each type of cancer and ulcer were assessed using Cox proportional hazards models, fitted using the Prentice pseudo-partial likelihood.^16^ Models were adjusted for age, sex, region (10 regions) and educational attainment (6 groups: no formal education, primary school, middle school, high school, technical school/college, university), and time in study was used as the time scale. Individuals were followed up until the first occurrence of each type of event and were censored if they died of other causes, or were lost to follow-up, or to January 1^st^, 2017, whichever occurred earlier. In the analysis of each subtype of gastric and oesophageal cancer respectively, individuals were followed up until the first occurrence of any gastric or oesophageal cancer respectively and other subtypes were censored. In the analysis of gastric and duodenal ulcer, individuals in the subcohort were censored at the time of any cancer diagnosis to match the selection criteria of the ulcer case groups, and individuals with a history of peptic ulcer at baseline were excluded. The analysis was repeated additionally adjusting for age^2^, smoking status (3 groups: never, ex-regular, occasional, current regular smoking), alcohol drinking (3 groups: never regular, ex-regular and current regular drinking) and body mass index (BMI). Adjusted hazard ratios (HRs) and 95% confidence intervals (CIs) for each outcome by sero-status for each pathogen (and each antigen), were estimated. The plausibility of the proportional hazards assumption was assessed using plots of scaled Schoenfeld residuals and the associated chi-squared tests.^17,18^

Analysis was done using R version 4.1.1^19^ and packages ‘survival’^20^ and ‘ckbplotr’.^21^

## Results

### Characteristics of individuals in the case-subcohort study

Among the 3945 participants that were included in the main analysis, the mean age at baseline of subcohort participants was lower than that of the cancer or ulcer cases (51.8 [standard deviation (sd) 10.8] and 58.5 [9.7], respectively). 61.7% of subcohort participants and 34.1% of cases were female. The proportions of participants living in an urban area were similar among subcohort participants and cases (50.2% and 44.5%, respectively). Proportions with at least 6 years of education and other socioeconomic factors (having a private toilet or fridge) varied between the different study arms. Subcohort participants and duodenal ulcer cases were more likely to have had at least 6 years of education than the other study arms. Prevalence of smoking and alcohol drinking among men varied by study arm and was highest among men with non-cardia gastric cancer and with oesophageal cancer (Table 1). Levels of adiposity were similar in subcohort participants and cases.

### Associations of pathogen seropositivity with cancers and ulcers

As shown in our previously published paper, seropositivity in the subcohort varied by pathogen, from 0.1% for HIV and HTLV to 99.8% for EBV, with low correlations between markers of different pathogens.^13^ Since seroprevalence of HIV and HTLV was too low for associations with outcomes to be reliably estimated and these pathogens are not included in the results. The seroprevalence of HPV-16, HHV-6, and HHV-7 was higher in women than in men. Seropositivity for HBV and HPV-16 increased with age, and urban residence was associated with a higher seroprevalence of *H. pylori*. There were no clear associations with ever-regular smoking or alcohol drinking.^13^

**Figure 1:**
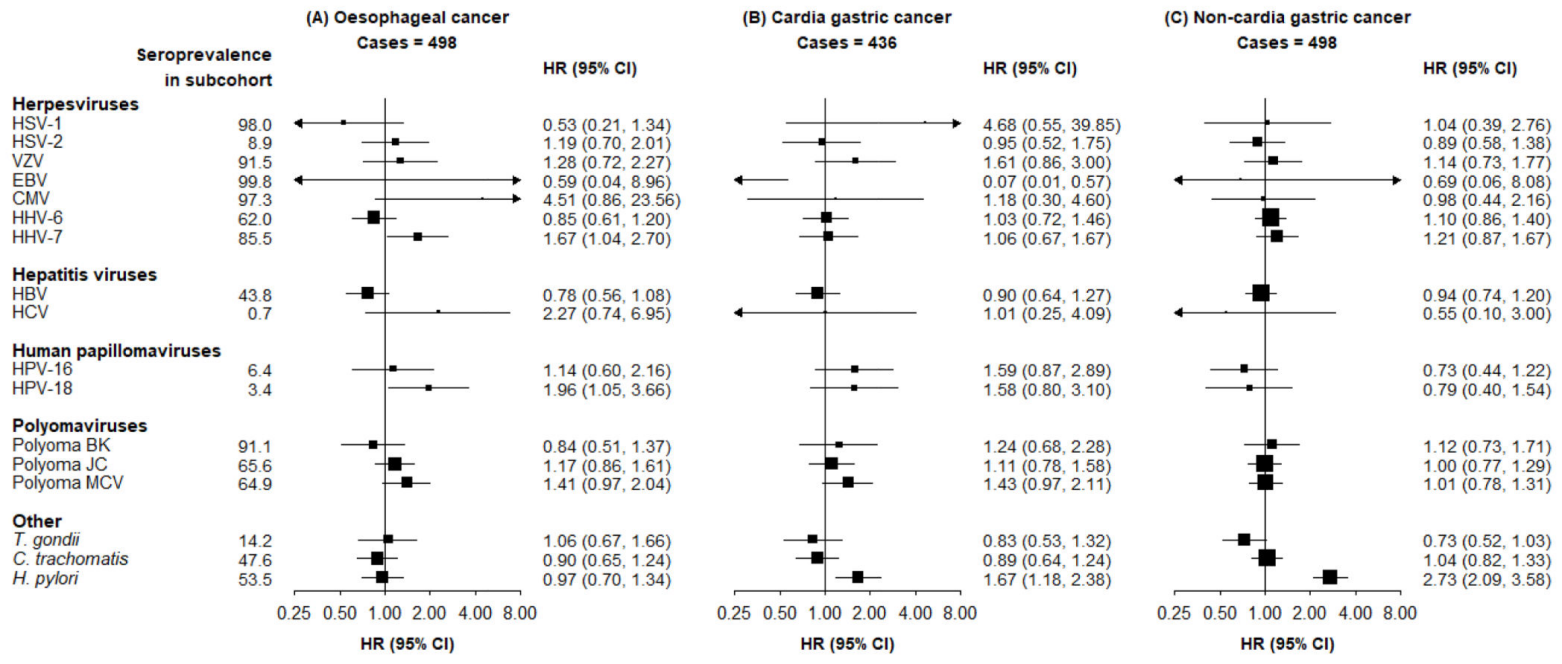
Adjusted HRs for risks of (A) oesophageal cancer, (B) gastric cardia cancer, and (C) non-cardia gastric cancer, associated with seropositivity to individual pathogens HRs are adjusted for age, sex, region and education. Squares are the estimated HRs and line segments their 95% CIs. The area of the square is inversely proportional to the variance of the logHR.

**Figure 2:**
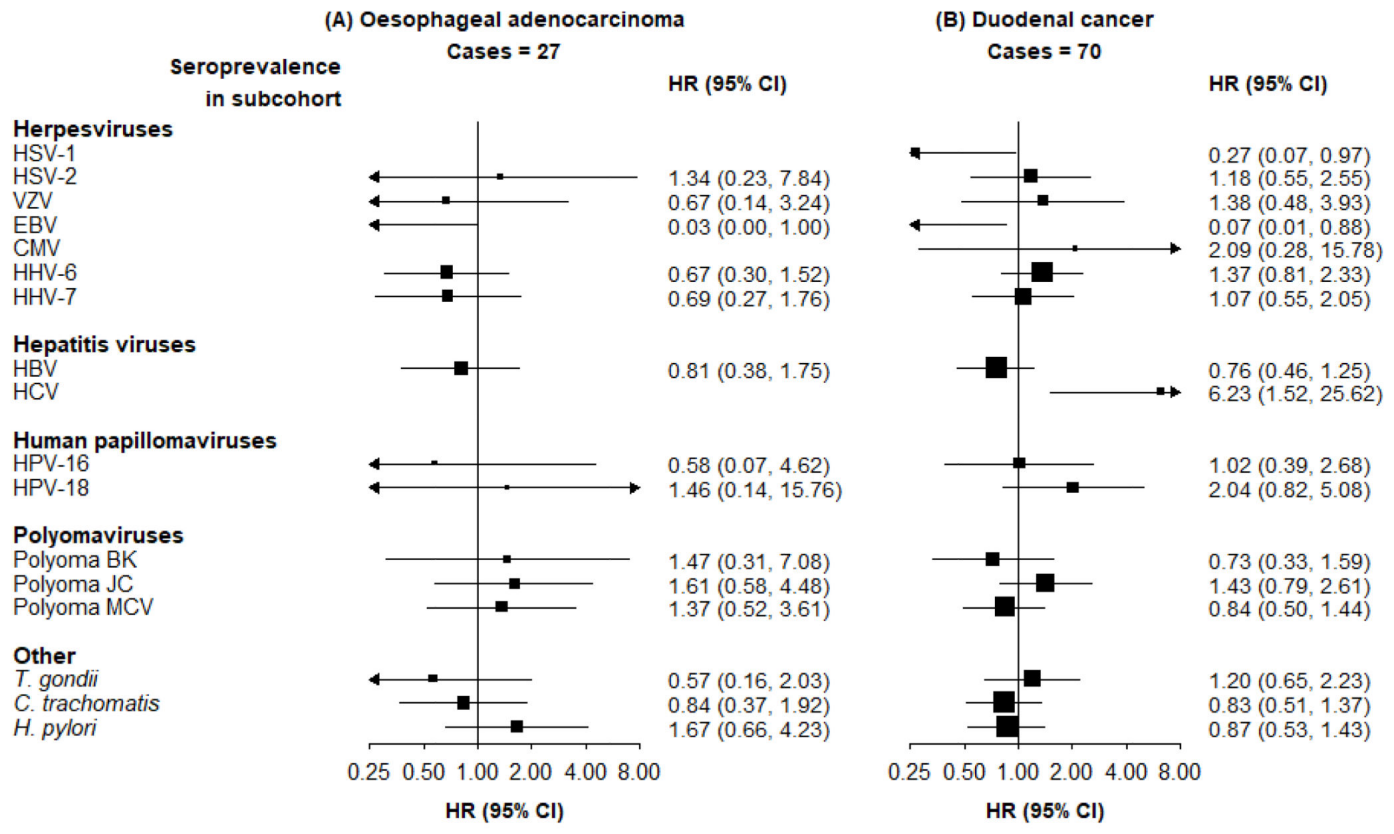
Adjusted HRs for risks of (A) oesophageal adenocarcinoma and (C) duodenal cancer, associated with seropositivity to individual pathogens HRs are adjusted for age, sex, region and education. Squares are the estimated HRs and line segments their 95% CIs. The area of the square is inversely proportional to the variance of the logHR.

Compared with seronegative participants, those seropositive for HHV-7 and HPV-18 had a higher risk of oesophageal cancer (adjusted HR 1.67 [95% CI 1.04-2.70] and 1.96 [1.05-3.66], respectively) (Figure 1). *H. pylori* infection was associated with a higher risk of NCGC (2.73 [2.09-3.58]) and CGC (1.67 [1.18-2.38]). More detailed results on the associations between *H. pylori* and gastric cancer have been reported separately as part of a meta-analysis.^9^ There was no association of *H. pylori* with oesophageal cancer, which is considered to largely consist of non-adenocarcinoma subtypes (0.97 [0.70-1.34]). There was no evidence of any association of seropositivity for any pathogen with risk of EAC, and precision of these estimates was low (Figure 2). HSV-1 was associated with a lower risk of duodenal cancer (0.27 [0.07-0.97]), and HCV was associated with a higher risk of duodenal cancer (6.23 [1.52-25.62]).

Inverse associations of EBV with some types of cancer were observed, but the precision of the estimates was low due to the small number of EBV-negative individuals. There was no evidence of any association between seropositivity for any of the polyomaviruses, *T. gondii*, or *C. trachomatis*, and any of the outcomes.

**Figure 3:**
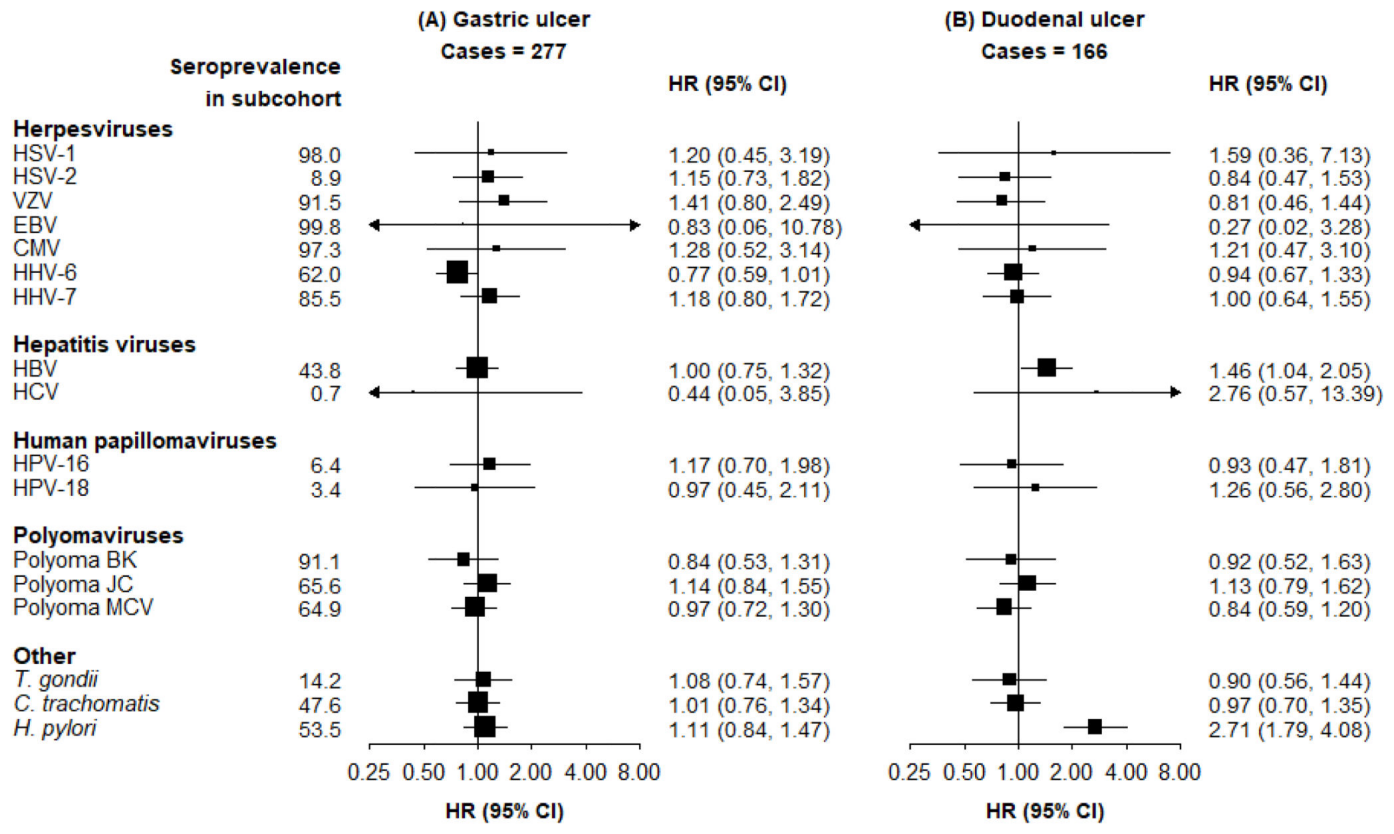
Adjusted HRs for risks of (A) gastric ulcer and (B) duodenal ulcer, associated with seropositivity to individual pathogens Individuals with a history of peptic ulcer at baseline are excluded. HRs are adjusted for age, sex, region and education. Squares are the estimated HRs and line segments their 95% CIs. The area of the square is inversely proportional to the variance of the logHR.

HBV and *H. pylori* were associated with a higher risk of duodenal ulcer (1.46 [1.04-2.05] and 2.71 [1.79-4.08], respectively). However, none of the pathogens were associated with gastric ulcer (Figure 3).

### Associations of antigen seropositivity with cancers and ulcers

The seroprevalence of antibodies to *H. pylori* antigens in the subcohort varied from 11.4% (HpaA) to 70.8% (CagA). Several *H. pylori* antibodies were associated with CGC and NCGC (eFigure 2) and duodenal ulcer (eFigure 3). Seropositivity for HPV-16 E7 was associated with a higher risk of non-cardia gastric cancer (1.43 [1.01-2.05]) (eFigure 4). There was no evidence of any association between individual antibodies for pathogens other than *H. pylori* and risk of non-cardia gastric cancer (eFigure 4). Seropositivity for EBV VCAp18 was inversely associated with risk of gastric cardia cancer (0.54 [0.32-0.92]). HCV NS3 was associated with a higher risk of duodenal cancer (4.20 [1.12-15.67]). HBc was associated with a higher risk of duodenal ulcer (1.45 [1.04-2.04]) (eFigure 5). The magnitude of the association of HBe with duodenal ulcer was similar (1.40 [0.99-1.96]).

There was no evidence against the proportional hazards assumption in any of the analyses. Associations of pathogens (eFigures 6 and 7) and antigens (eFigures 8-11) with cancers and ulcers did not change substantially with additional adjustment in sensitivity analyses.

## Discussion

In this prospective study of multiple pathogens and risks of gastrointestinal tract cancers and ulcers among Chinese adults, we found that *H. pylori* seropositivity was associated with risks of developing NCGC, CGC, and duodenal ulcer. Hepatitis viruses were associated with higher risks of duodenal ulcer and cancer, with a 46% higher risk of duodenal ulcer with HBV infection and a six-fold higher risk of duodenal cancer with HCV infection. HHV-7 seropositivity was associated with ~67% higher risk of oesophageal cancer. No other pathogens were associated with the GI cancers and ulcers studied.

Although there was a lower seroprevalence of *H. pylori* in our study compared with prior studies in East Asians, the magnitude of its association with NCGC was remarkably consistent across studies in East Asians,^14,15,22^ and stronger than that in Western populations.^23^ For example, *H. pylori* infection was associated with an OR of 2.80 (2.25-3.48) for NCGC in a pooled analysis of 8 prospective studies in East Asia (1608 cases),^15^ and with an OR of 1.90 (1.01-3.57) in MCC-Spain multicentre case-control study (202 cases).^23^ Previously published findings on the potential association between *H. pylori* and CGC have varied widely.^9,23,24^ To date, two case-control studies using multiplex serology have been conducted. *H. pylori* seropositivity was not associated with CGC in studies in Iran (142 cases; OR 1.70, 0.60-4.81) ^24^ or in Spain (61 cases; OR 0.54, 0.25-1.15).^23^ Using the same multiplex serology assay, the present study found a significantly higher risk of CGC in Chinese with an *H. pylori* infection. This discrepancy may partly be due to the small number of cases included in the previous studies (CKB with >2-fold as many cases as in all the two studies combined), blood samples collected near cancer diagnosis, and also be attributed to the fact that there are two distinct aetiologies of CGC.^25^ One type resembles NCGC, being a consequence of atrophic gastritis due to *H. pylori* infection and concentrating in East Asians. Another type resembles oesophageal adenocarcinoma (EAC), associated with gastrooesophageal reflux and mainly involved in Western populations. We did not observe a significantly higher risk of EAC associated with *H. pylori* infection, the estimates were, however, numerically similar to those for CGC, which may reflect this hypothesis of common pathophysiology between EAC and CGC. We found an association of *H. pylori* infection with duodenal ulcer, as expected, but not with gastric ulcer. This may be because *H. pylori* usually colonizes the antrum, which then leads to hypersecretion of acid, predisposing to duodenal ulcers.^26^

The role of HBV infection in the development of hepatocellular carcinoma is well-established. As HBV infection also exists in gastric mucosa epithelial cells, it may be possible that HBV infection increases the risk of gastric cancer in a similar mechanism of HBV-related hepatocellular carcinoma,^27^ or related to altered immune control. However, few epidemiological studies have shown conflicting results regarding the association between HBV infection and gastric cancer.^28^ A previous study using the CKB showed that hepatitis B surface antigen (HBsAg) was associated with an HR of 1.41 (1.11-1.80) for stomach cancer (2157 cases), and the association was further replicated in two other small Chinese studies using polymerase chain reaction or immunohistochemistry test measurements of HBsAg.^29^ In the present study, we measured HBV e antibody and core antibody levels and both were associated with higher risk of duodenal ulcer. HCV is also an established cause of hepatocellular carcinoma^30^ and has been previously shown to be associated with a range of gastrointestinal and other cancers.^31^ The present study found that HCV infection was associated with a 6-fold higher risk of duodenal cancer, but the 95% confidence interval was wide due to the small number of cases and low prevalence of HCV. Certainly, the roles of HBV or HCV in gastric cancer development merit further investigations.

The oncogenic potential of HPV is well known in the context of cervical carcinoma, but its role in the development of oesophageal cancer remains controversial. A recent meta-analysis involving 33 case-control studies worldwide suggested that HPV infection was associated with risk of oesophageal cancer (OR=1.62, 1.33-1.98).^32^ However, the mechanistic evidence using tumour tissue does not support the etiological role of HPV in the ESCC carcinogenesis.^33^ The E6 and E7 genes of the high-risk HPV types encode oncoproteins, and both act by interfering with the activity of cellular tumour suppressor proteins.^34^ Epidemiological and mechanistic evidence on the causative role of HPV in NCGC is unclear. In the only previous prospective study involving only 70 NCGC cases, HPV-16 infection determined by ELISA was not associated with risk of NCGC (OR 0.4, 0.1-1.6).^35^ Overall, the present study is the first to explore the association of HPV and related oncogenic proteins with gastric cancer using multiplex serology measurements.

In this study, HHV-7 infection was reported for the first time to be associated with a higher risk of oesophageal cancer. Several factors including immunosuppressive properties and pro-inflammatory properties indicating by their ability to alter the cytokine expression profile of infected cells suggest a possible role for HHV-7 in the pathogenesis of cancer.^36^ However our findings should be interpreted with caution as multiple pathogens were assessed and some associations may be observed by chance. If a false discovery rate correction is considered, only the association of H. pylori with NCGC and duodenal ulcer would be considered significant.

A strength of the present study is its prospective design, with exclusion of the first two years after blood collection to limit reverse causation, allowing estimation of associations between seropositivity of pathogens and future risk of disease. Moreover, we included well-characterised cases of different subtypes of cancers of adjacent sites and precursor lesions, which allows the assessment of relationships of infections with different stages of the underlying process of cancer development and allows comparison of associations with cancer subtypes to explore the extent to which there is common aetiology. The use of a multiplex serology assay enabled the measurement of several antigens and the definition of seropositivity to several important pathogens. The availability of detailed participant characteristics allowed adjustment for potential confounders; however residual confounding may still exist, including from infections by other unmeasured pathogens. Another limitation of the study is the small number of cases for some disease subtypes, despite the large cohort from which they were identified, because of their low incidence in the population.

## Conclusion

In summary, in this prospective study of infections and risk of cancer and its precursors in China, we found associations between various pathogens and disease subtypes. In particular, HCV was associated with a higher risk of duodenal cancer, and *H. pylori* and several of its specific antigens were associated with CGC and NCGC and with duodenal ulcer. Further studies are needed to replicate these associations and to explore the potential mechanisms involved.

## Supplementary Material

Supplementary File

## Figures and Tables

**Table 1:** Baseline characteristics of the subcohort and cases.

	Subcohort (n=1986)	Cases (n=1964)	EC (n=498)	EAC (n=27)	CGC (n=436)	NCGC (n=497)	DC (n=70)	GU (n=197)	DU (n=297)
Mean age, years	51.8	58.5	60.7	62.4	61.1	59.0	56.9	54.2	52.9
Female	61.7	34.1	26.9	25.9	25.5	34.4	44.3	49.8	42.1
Urban region	50.2	44.5	25.3	44.4	36.7	70.4	58.6	30.0	60.9
Education ≥6 years	51.8	38.6	28.5	40.7	32.6	45.1	45.7	38.0	59.9
Household size	3.7	3.7	3.6	3.6	3.7	3.5	3.8	4.0	3.8
Private toilet	55.7	42.7	24.5	29.6	33.7	62.6	65.7	41.1	53.8
Fridge	57.9	50.7	40.6	48.1	45.6	66.0	70.0	40.4	61.4
Ever regular smoking									
Males	74.1	81.3	84.1	80.0	76.0	87.4	71.8	79.9	76.3
Females	3.6	4.8	4.5	0	0	4.1	3.2	8.1	7.2
Current regular alcohol intake									
Males	33.8	37.1	45.9	30.0	27.4	41.1	28.2	31.5	34.2
Females	2.8	0.9	0.7	0	0	1.2	3.2	1.4	0
Mean BMI, kg/m^2^	23.8	23.5	23.1	24.0	24.0	23.6	24.4	23.2	23.3
**Daily consumption**									
Meat	31.8	25.7	17.9	25.9	16.1	35.0	28.6	24.2	42.1
Preserved vegetables	17.7	22.3	18.7	29.6	24.3	32.2	11.4	14.5	17.3
Fresh fruit	22.0	14.0	7.4	18.5	7.6	24.9	18.6	11.1	18.8
Blood transfusions	4.0	4.9	5.0	3.7	5.0	4.4	5.7	4.0	7.1
HBV surface antigen positive	3.2	2.7	2.0	0	3.2	3.9	1.5	1.7	3.1
History of peptic ulcer	3.8	6.8	4.0	3.7	5.3	6.2	2.9	9.1	16.8
Treatment of peptic ulcer*	15.8	25.4	15.0	0	30.4	25.8	0	33.3	18.2

Numbers are percentages (within arm), unless otherwise specified. EC: oesophageal cancer; EAC: oesophageal adenocarcinoma; CGC: cardia gastric cancer; NCGC: noncardia gastric cancer; DC: duodenal cancer; GU: gastric ulcer; DU: duodenal ulcer.

* Percentages receiving treatment for peptic ulcer at baseline among participants with a history of peptic ulcer.

## Data Availability

Current study is based on the China Kadoorie Biobank (CKB). For open access data please visit https://www.ckbiobank.org/data-access. For the raw data from CKB that is utilized in this paper should contact ckbaccess@ndph.ox.ac.uk. Further information is available from the corresponding author upon request.

## Novelty and impact

Approximately 13% of cancers worldwide have been attributed to infections with certain pathogens, including *Helicobacter pylori (H. pylori)*, Human papillomaviruses (HPV) and Hepatitis viruses B and C.

This is the first study that investigated the role of 19 infectious pathogens and 43 antigens in the risk of different types of GI cancers/ulcers. In this large Chinese cohort, *H. pylori* seropositivity was associated with a higher risk of gastric non-cardia and cardia, and duodenal but not gastric ulcer. We also found an association of HCV with duodenal cancer and HBV with duodenal ulcer.

This study provides additional evidence supporting eradication of *H. pylori* for cancer prevention and has identified a new association which needs to be investigated in future research.

## List of abbreviations

BMI: body mass index
*C. trachomatis*: *Chlamydia trachomatis*
CGC: cardia gastric cancer
CI: confidence interval
CKB: China Kadoorie Biobank
CMV: cytomegalovirus
EAC: oesophageal adenocarcinoma
EBV: Epstein−Barr virus
*H. pylori*: *Helicobacter pylori*
HBsAg: hepatitis B surface antigen
HBV: hepatitis virus B
HCV: hepatitis virus C
HHV-6: human herpesvirus 6
HHV-7: human herpesvirus 7
HIV: human immunodeficiency virus
HPV: human papillomavirus
HPV-16: human papillomavirus 16
HPV-18: human papillomavirus 18
HR: hazard ratio
HSV-1: herpes simplex virus 1
HSV-2: herpes simplex virus 2
HTLV-1: human T lymphotropic virus type 1
MFI: median fluorescence intensity
NCGC: non-cardia gastric cancer
sd: standard deviation
*T. gondii*: *Toxoplasma gondii*
VZV: varicella zoster virus
