# Supplementary material for: Infectious pathogens and risk of oesophageal, gastric and duodenal cancers and ulcers in China: a case-cohort study
Source: Int J Cancer. Author manuscript; Available in PMC 2024 Apr 15. (PMC7615747; doi:10.1002/ijc.34814)

**Supplementary material**

**Infectious pathogens and risk of oesophageal, gastric and duodenal cancers and ulcers in China: a case-subcohort study**

Christiana Kartsonaki^1,2*^, Pang Yao^1*^, Julia Butt^3^, Rima Jeske^3^, Catherine de Martel^4^, Martyn Plummer^5^, Yu Guo^6^, Sarah Clark^1,2^, Robin G. Walters^1,2^, Yiping Chen^1,2^, Jun Lv^7^, Canqing Yu^7^, Michael Hill^1,2^, Richard Peto^1^, Liming Li^6^, Tim Waterboer^3^*^#^*, Zhengming Chen^1,2^*^#^*, Iona Y. Millwood^1,2^*^#^*, Ling Yang^1,2^*^#^*

**Joint first authors; ^#^Joint senior authors*

1. Clinical Trial Service Unit & Epidemiological Studies Unit (CTSU), Nuffield Department of Population Health, University of Oxford, Oxford, UK
2. Medical Research Council Population Health Research Unit (MRC PHRU), Nuffield Department of Population Health, University of Oxford, Oxford, UK
3. Infections and Cancer Epidemiology Division, German Cancer Research Center (DKFZ), Heidelberg, Germany
4. Early Detection, Prevention and Infections Branch, International Agency for Research on Cancer, Lyon, France
5. Department of Statistics, University of Warwick, Coventry, UK
6. Chinese Academy of Medical Sciences, Dong Cheng District, Beijing, China
7. Department of Epidemiology and Biostatistics, School of Public Health, Peking University Health Science Centre, Beijing, China

**Address for correspondence**

| Iona Y. Millwood  MRC PHRU & CTSU  Nuffield Department of Population Health,  Big Data Institute Building  University of Oxford,  Old Road Campus, Oxford, OX3 7LF, United Kingdom  Email: [iona.millwood@ndph.ox.ac.uk](mailto:iona.millwood@ndph.ox.ac.uk)  Tel: +44 (0) 1865 743612 | Ling Yang  MRC PHRU & CTSU  Nuffield Department of Population Health,  Big Data Institute Building  University of Oxford,  Old Road Campus, Oxford, OX3 7LF, United Kingdom  Email: [ling.yang@ndph.ox.ac.uk](mailto:ling.yang@ndph.ox.ac.uk)  Tel: +44 (0) 1865 743936 |
| --- | --- |

**9 May 2023**

**eTable 1. List of pathogens and antigens, cutoffs for antigen seropositivity and criteria for seropositivity of individual pathogens**

| **Pathogen** | **Antigen** | **Cutoff** | **Criteria for pathogen seropositivity** |
| --- | --- | --- | --- |
| **Herpesviruses** |  |  |  |
| HSV-1 (HHV-1) | gG | 54 |  |
| HSV-2 (HHV-2) | mgG unique | 180 |  |
| VZV (HHV-3) | gE/gI | 100 |  |
| EBV (HHV-4) | Zebra | 74 | ≥2 positive out of 4 |
|  | EA-D | 110 |  |
|  | VCAp18 | 2526 |  |
|  | EBNA | 411 |  |
| CMV (HHV-5) | pp150 N | 655 | ≥2 positive out of 3 |
|  | pp52 | 1101 |  |
|  | pp28 | 200 |  |
| HHV-6 | IE1A | 300 | ≥1 positive out of 2 |
|  | IE1B | 300 |  |
| HHV-7 | U14 | 100 |  |
| **Hepatitis viruses** |  |  |  |
| HBV | HBc | 1000 | 2 positive out of 2 |
|  | HBe | 1000 |  |
| HCV | Core | 300 | 2 positive out of 2 |
|  | NS3 | 150 |  |
| **HIV** | gag | 1000 | 2 positive out of 2 |
|  | env | 150 |  |
| **HTLV** | gag | 700 | 2 positive out of 2 |
|  | env | 50 |  |
| **Human papillomaviruses** |  |  |  |
| HPV-16 | L1 | 80 |  |
| HPV-16 | E6 | 100 |  |
|  | E7 | 50 |  |
| HPV-18 | L1 | 170 |  |
| **Polyomaviruses** |  |  |  |
| BKV | VP1 | 250 |  |
| JCV | VP1 | 250 |  |
| MCV | VP1 | 250 |  |
| ***C. trachomatis*** | Pgp3 | 300 |  |
| ***T. gondii*** | sag1 | 50 | ≥1 positive out of 2 |
|  | p22 | 50 |  |
| ***H. pylori*** | HP0243-NapA | 120 | ≥4 positive out of 12 |
|  | HP0305 | 130 |  |
|  | HP0410-HpaA | 200 |  |
|  | HP0547-CagA (N+C) | 1500 |  |
|  | HP0695-HyuA (C) | 280 |  |
|  | HP0010-GroEL | 500 |  |
|  | HP0073-UreaseA | 300 |  |
|  | HP0875-Catalase | 300 |  |
|  | HP0887-VacA (N+C) | 385 |  |
|  | HP1098 | 150 |  |
|  | HP1104-Cad | 100 |  |
|  | HP1564 | 150 |  |

**eFigure 1. Flow diagram of study design and participant selection**

**
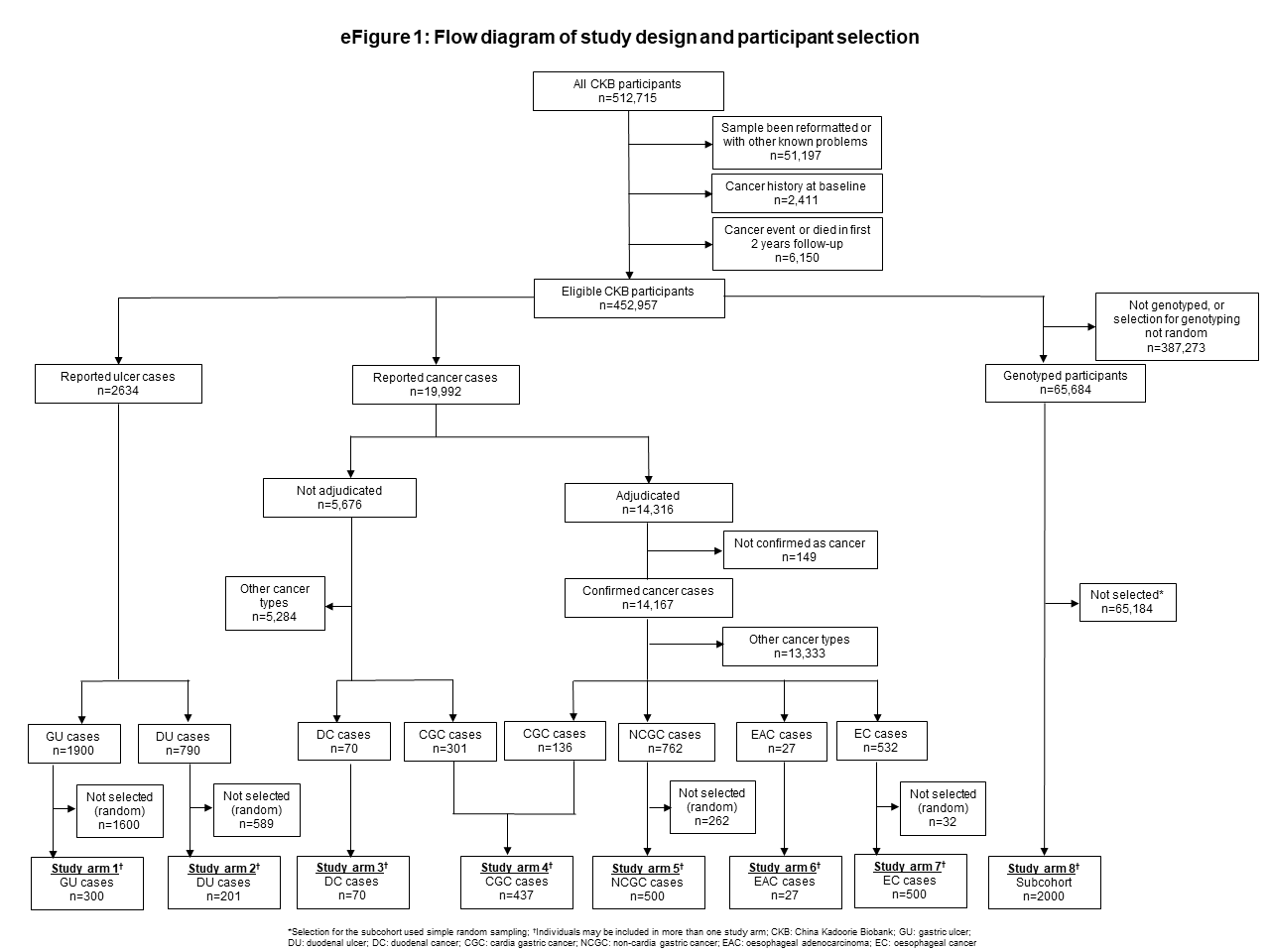
**

**eFigure 2. Adjusted HRs for risks of (A) oesophageal cancer, (B) oesophageal adenocarcinoma, (C) cardia gastric cancer, (D) non-cardia gastric cancer, and (E) duodenal cancer, associated with seropositivity of individual *H. pylori* antibodies**

HRs are adjusted for age, sex, region and education

**
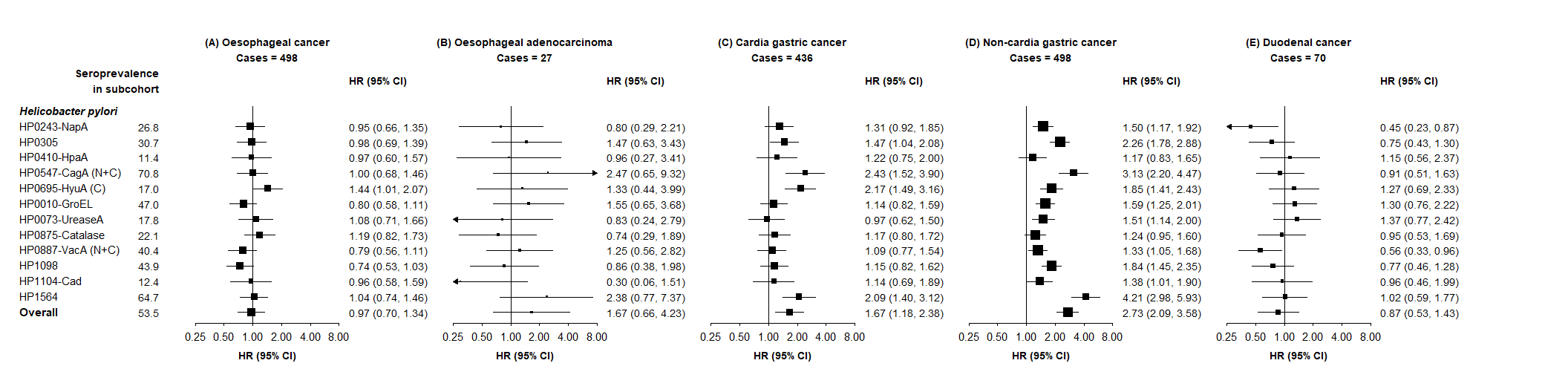
**

**eFigure 3. Adjusted HRs for risks of (A) gastric and (B) duodenal ulcer, associated with seropositivity of individual *H. pylori* antibodies**

HRs are adjusted for age, sex, region and education
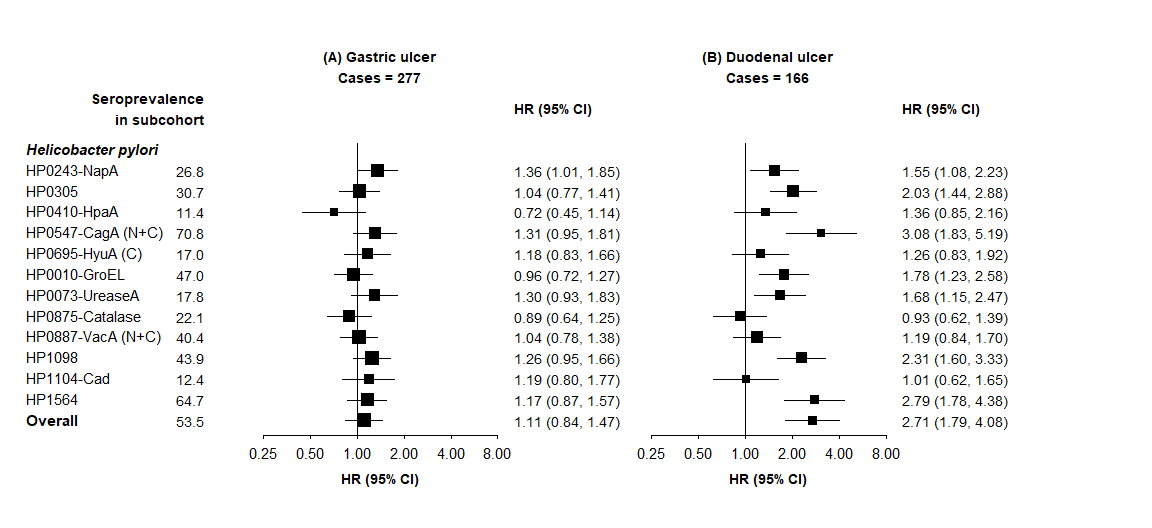


**eFigure 4. Adjusted HRs for risks of (A) oesophageal cancer, (B) oesophageal adenocarcinoma, (C) cardia gastric cancer, (D) non-cardia gastric cancer, and (E) duodenal cancer, associated with seropositivity of individual antibodies**

HRs are adjusted for age, sex, region and education**
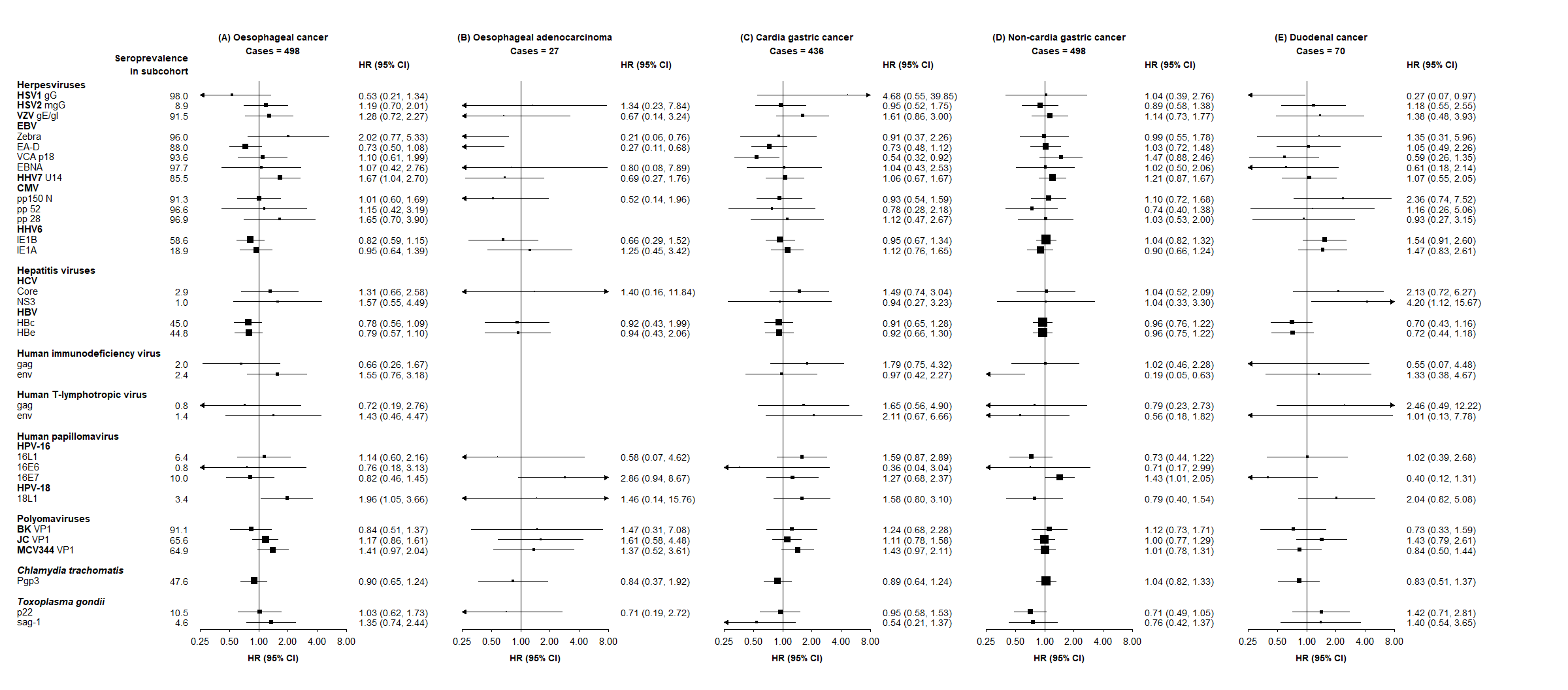
**

**eFigure 5. Adjusted HRs for risks of (A) gastric and (B) duodenal ulcer, associated with seropositivity of individual antibodies**

HRs are adjusted for age, sex, region and education**
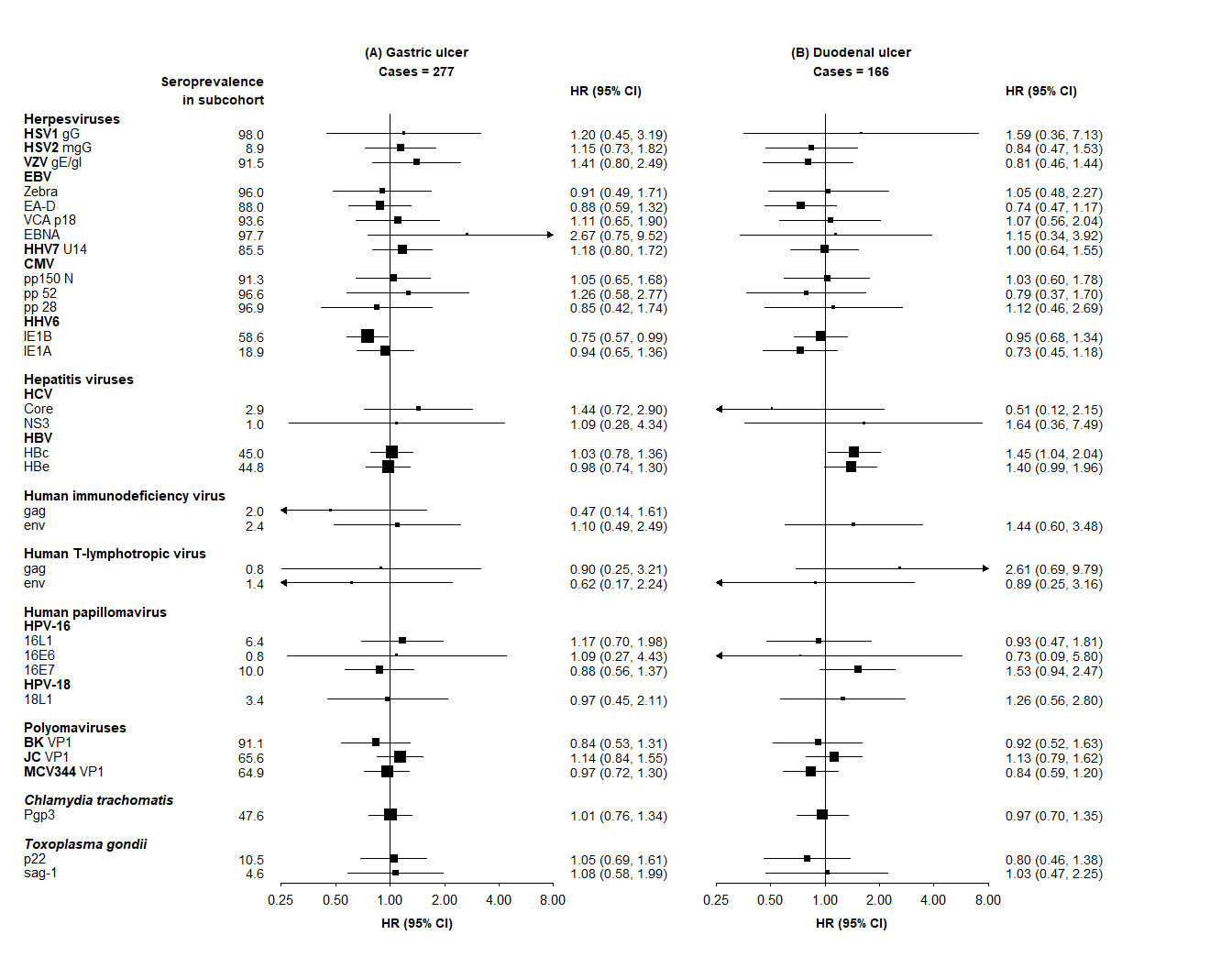
**

**eFigure 6. Adjusted HRs for risks of (A) oesophageal cancer, (B) oesophageal adenocarcinoma, (C) cardia gastric cancer, (D) non-cardia gastric cancer, and (E) duodenal cancer, associated with seropositivity of individual pathogens**

HRs are adjusted for age, sex, region, education, age^2^, smoking status, alcohol consumption and BMI


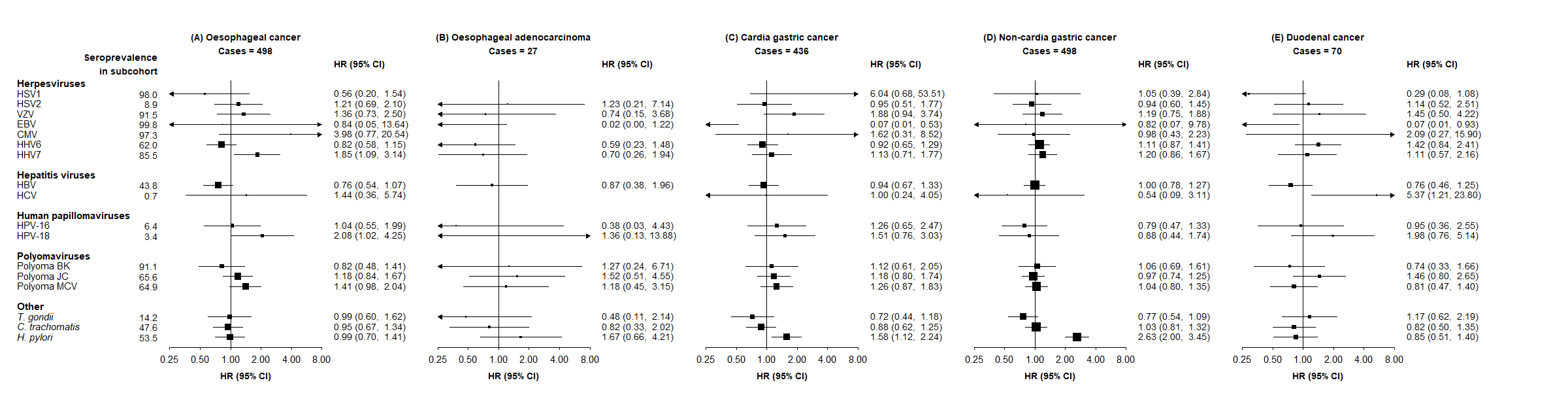


**eFigure 7. Adjusted HRs for risks of (A) gastric and (B) duodenal ulcer, associated with seropositivity of individual pathogens**

HRs are adjusted for age, sex, region, education, age^2^, smoking status, alcohol consumption and BMI
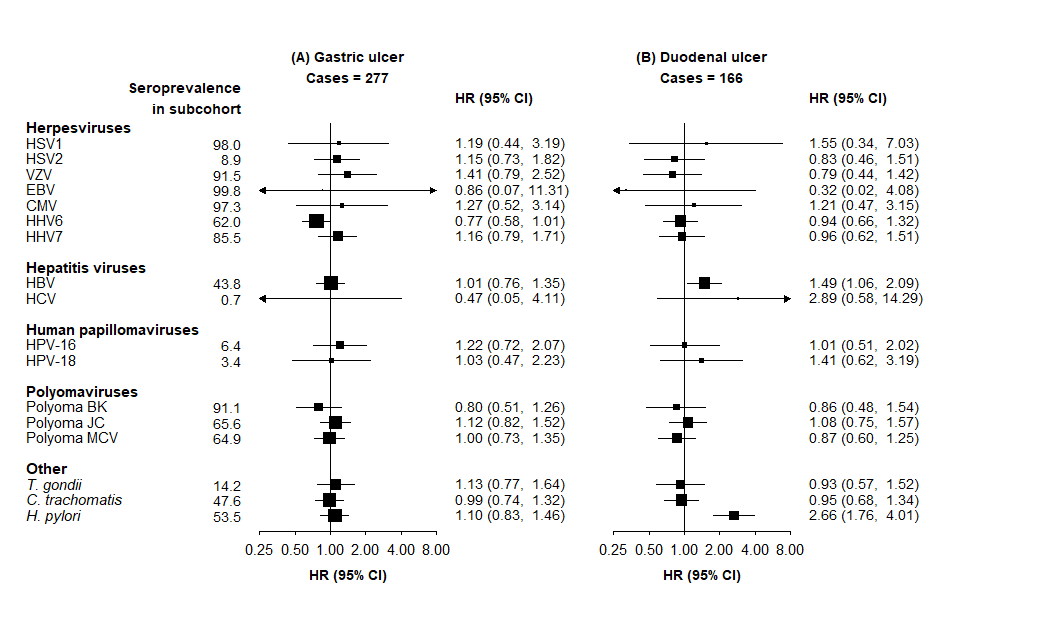


**eFigure 8. Adjusted HRs for risks of (A) oesophageal cancer, (B) oesophageal adenocarcinoma, (C) cardia gastric cancer, (D) non-cardia gastric cancer, and (E) duodenal cancer, associated with seropositivity of individual *H. pylori* antibodies**

HRs are adjusted for age, sex, region, education, age^2^, smoking status, alcohol consumption and BMI

**
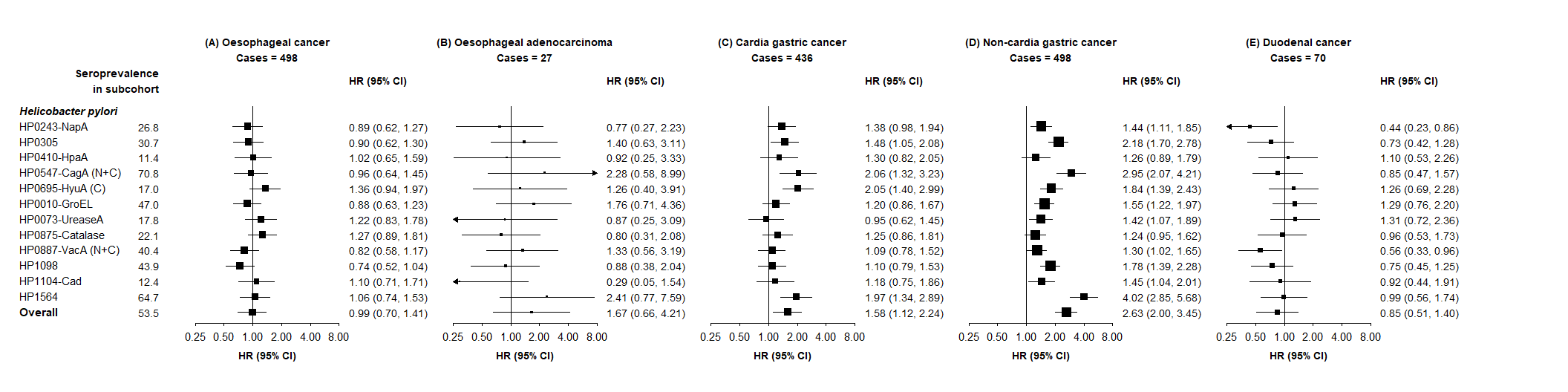
**

**eFigure 9. Adjusted HRs for risks of (A) oesophageal cancer, (B) oesophageal adenocarcinoma, (C) cardia gastric cancer, (D) non-cardia gastric cancer, and (E) duodenal cancer, associated with seropositivity of individual *H. pylori* antibodies**

HRs are adjusted for age, sex, region, education, age^2^, smoking status, alcohol consumption and BMI**
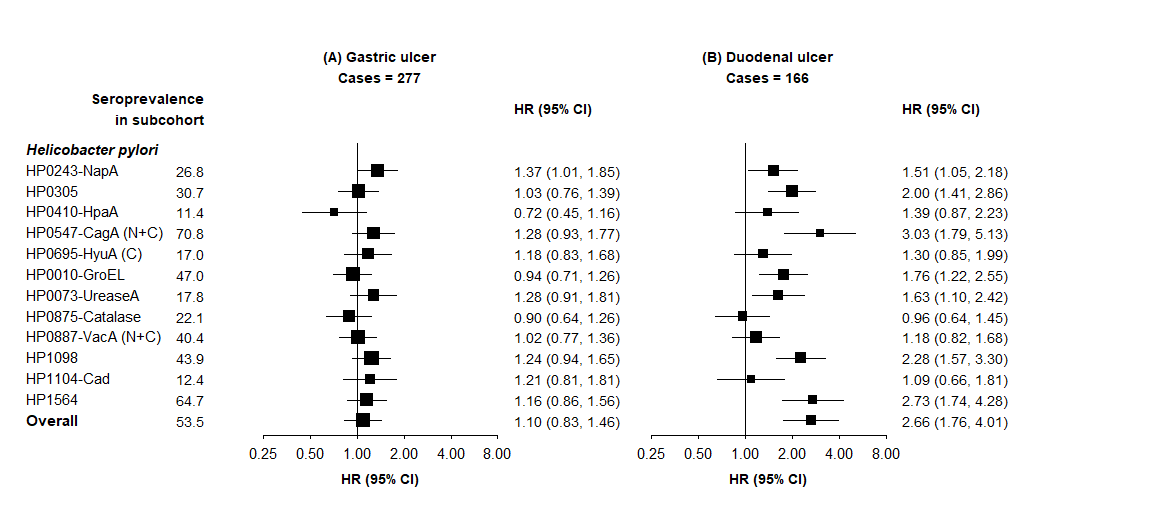
**

**eFigure 10. Adjusted HRs for risks of (A) oesophageal cancer, (B) oesophageal adenocarcinoma, (C) cardia gastric cancer, (D) non-cardia gastric cancer, and (E) duodenal cancer, associated with seropositivity of individual antibodies**

HRs are adjusted for age, sex, region, education, age^2^, smoking status, alcohol consumption and BMI**
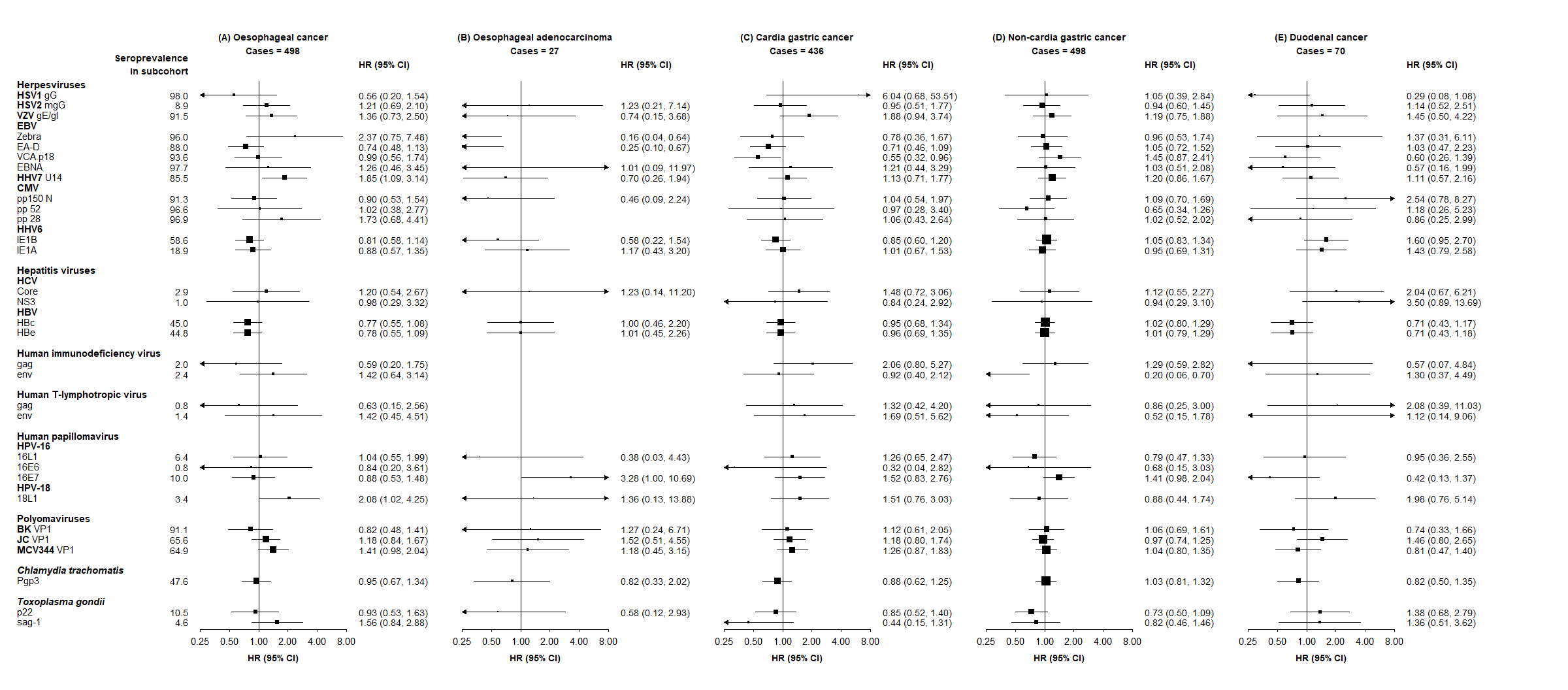
**

**eFigure 11. Adjusted HRs for risks of (A) gastric and (B) duodenal ulcer, associated with seropositivity of individual antibodies**

HRs are adjusted for age, sex, region, education, age^2^, smoking status, alcohol consumption and BMI
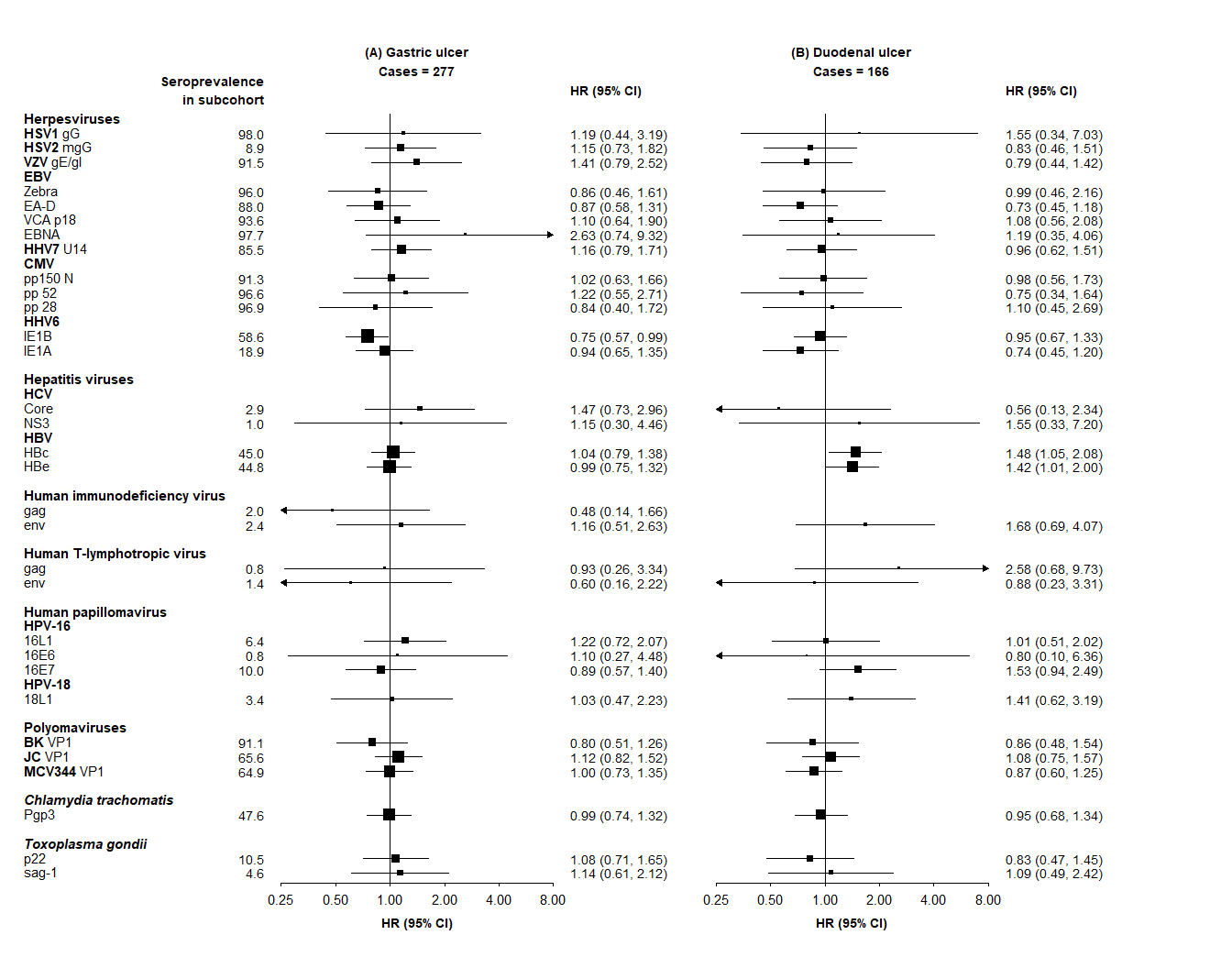

Supplement: Supplementary File [file EMS194687-supplement-Supplementary_File.docx]
